# Lactulose selectively stimulates members of the gut microbiota, as determined by multi-modal activity-based sorting

**DOI:** 10.1080/19490976.2025.2525482

**Published:** 2025-06-27

**Authors:** Hamid Rasoulimehrabani, Alessandra Riva, Deniz Inan, Adnan Hodžić, Bela Hausmann, Georgi Nikolov, Sanaz Khadem, Norbert Hieger, Julia Wiesenbauer, Christina Kaiser, Verena Filz, Thomas Böttcher, David Berry

**Affiliations:** a Center for Microbiology and Environmental Systems Science, Department of Microbiology and Ecosystem Science, University of Vienna, Vienna, Austria; b Doctoral School in Microbiology and Environmental Science, University of Vienna, Vienna, Austria; c Chair of Nutrition and Immunology, TUM School of Life Sciences, Technical University of Munich, Freising, Germany; d Joint Microbiome Facility of the Medical University of Vienna and The University of Vienna, Vienna, Austria; e Department of Laboratory Medicine, Medical University of Vienna, Vienna, Austria; f Department of Biological Chemistry, University of Vienna, Vienna, Austria

**Keywords:** Raman microspectroscopy, prebiotics, BONCAT, RACS, keystone species

## Abstract

There is much interest in the development of dietary supplements that selectively promote the growth of beneficial gut bacteria. The selectivity of many candidate prebiotics has, however, not been thoroughly investigated. Here, we evaluated stimulation of the human gut microbiota by the disaccharide lactulose using an *ex vivo* multimodal activity-based cell sorting approach. Incubation of human donor stool with lactulose resulted in growth or stimulation of a restricted diversity of bacterial genera, most prominently *Bifidobacterium*, *Collinsella*, and *Lactococcus*. Physiological analysis of lactulose-responsive strains isolated by Raman activated cell sorting revealed that most were capable of lactulose degradation. Among these isolates, *Lactococcus lactis* could not degrade lactulose, but its growth was boosted by co-cultivation with lactulose degraders. This suggests that inter-species facilitation contributes to the lactulose degradation niche. Moreover, we observed that lactulose selectively activates metabolically important taxa, including health-associated genera such as *Faecalibacterium* and *Gemmiger*^1,2,3^, potentially indicating broader functional effects beyond compositional changes. These results provide novel insights into the physiology and ecology of lactulose utilization by the human gut microbiota and underscore the potential of lactulose as a prebiotic dietary supplement.

## Introduction

The human gastrointestinal tract hosts a diverse community of microorganisms known as the gut microbiota that plays important roles in multiple aspects of human physiology, including digestion and nutrition, metabolism, and immune and neurological function.^1,2^ The microbiota is particularly dense and diverse in the large intestine, where it is dominated by the bacterial phyla *Bacillota, Bacteroidota, Actinomycetota*, and *Pseudomonadota*.^3^ The composition of the gut microbiota is influenced by several factors, with diet being an important determinant.^4,5^ Diets rich in non-digestible polysaccharides have been shown to increase the abundance of beneficial gut microbes, particularly members of *Bacillota* and *Actinomycetota*.^6^ Additionally, supplementation of diet with non-digestible dietary polysaccharides such as inulin, fructooligosaccharides (FOS), and galactooligosaccharides has been reported to support the growth of taxa such as *Bifidobacterium* and *Lactobacillus*.^7,8^ This has led to the concept of prebiotics, which are specific dietary supplements that selectively promote the growth of certain gut microorganisms, modulate microbiota function, and confer a health benefit to the host.^9–12^ However, for many candidate prebiotics, it remains unclear which specific taxa are stimulated and how their metabolic interactions influence gut microbiome dynamics.^3^

Among candidate prebiotics, lactulose has emerged as a promising yet underexplored synthetic disaccharide, composed of fructose and galactose linked by a β-1,4-glycosidic bond.^13,14^ It is primarily used in medicine for treating constipation, hepatic encephalopathy, and diabetes.^13,15,16^ Recent *in vitro* and *in vivo* studies have indicated that lactulose at low doses may also have prebiotic effects^17^ and can increase the intestinal levels of *Bifidobacterium* and *Lactobacillus*,^18^ which may help in maintaining gut health by boosting production of short-chain fatty acids (SCFAs) and inhibiting enteric pathogens.^13,14^ In both human and mouse models, oral intake of lactulose increased intestinal *Bifidobacterium* and acetate production, resulting in gut lumen acidification that inhibited antibiotic-resistant bacteria and was associated with reduced infection rates and improved liver disease outcomes.^19^ In the present study, we examined the stimulatory effect of lactulose on the gut microbiota of healthy human adults in *ex vivo* incubations. We employed a multi-modal activity-based sorting approach to identify which microbial cells were metabolically active under the applied incubation conditions. Our findings were further validated through physiological analysis and co-cultivation assays, which revealed that lactulose not only promoted the growth and metabolic activity of a subset of gut bacteria, but also enabled ecological facilitation within the microbiota. These results highlight lactulose’s role in shaping community structure by selectively stimulating primary degraders such as *Bifidobacterium adolescentis* and *Collinsella aerofaciens*, which in turn facilitated the growth of non-degraders like *Lactococcus lactis*.

## Results

### Lactulose is rapidly degraded by the gut microbiota

**Figure 1.**
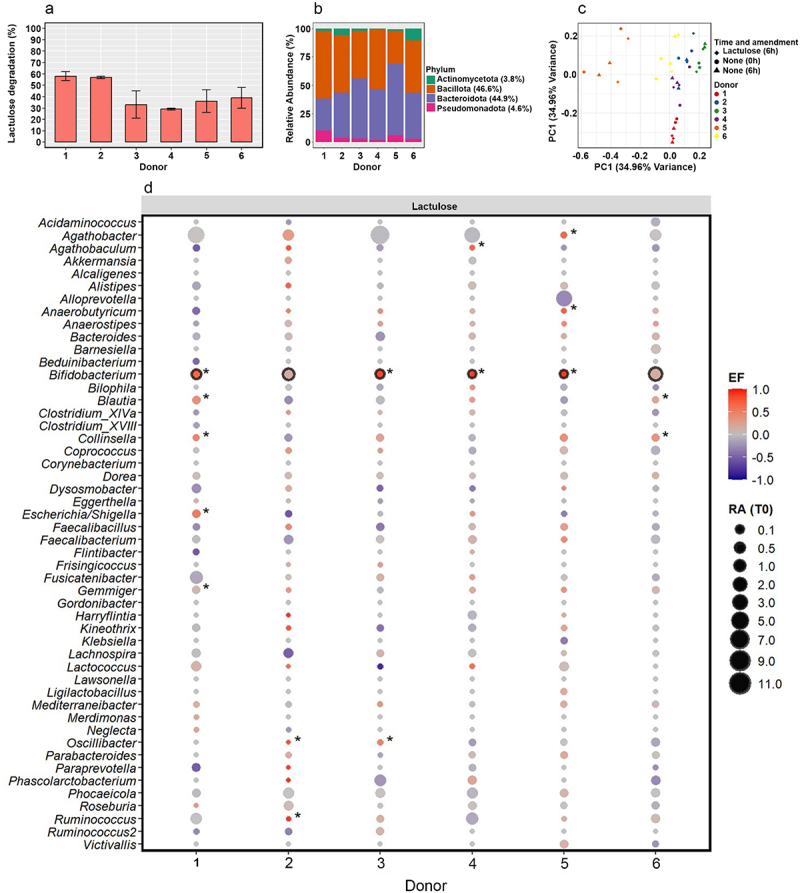
Lactulose degradation and gut microbiome composition. (a) Lactulose degradation (%) after 6h of anaerobic incubation with fresh fecal samples from 6 donors (mean ± SD: 41.5% ± 12.5, *n* = 12) measured with HPAEC. The bar represents the mean of two technical replicates and error bars indicate the standard deviation. (b) Phylum-level composition (relative abundance, as %) of fecal samples prior to incubation (*t* = 0 h). The mean of technical replicates is shown. The mean of relative abundances across the sample set are listed in the legend. (c) Principal coordinate analysis (PCoA) ordination of genus-level microbiome profiles for lactulose-amended samples at 6 hours and no amendment samples at 0 and 6 hours. Technical replicates are shown. (d) Change in relative abundance of abundant bacterial genera after 6 h incubation of lactulose-amended samples. Bubble size indicates relative abundance (RA) at 0h. The change in relative abundance during incubation, calculated as a normalized and scaled enrichment factor (EF; see materials and methods), is indicated by bubble color. Genera significantly enriched in individual donors are marked with an asterisk and those significantly enriched across all six donors (as determined by DESeq2, Wald Test, *p*<0.05, *n* = 36 samples) are outlined in black.

We employed a multi-modal activity-based sorting approach to identify gut microbes that are stimulated by lactulose.^20^ Freshly collected fecal samples from six healthy adults were incubated anaerobically for 6 h with lactulose or its component monosaccharides, galactose and fructose, in the presence of the cellular activity markers D₂O and L-azidohomoalanine (AHA; Supplementary Figure S1). 6 h incubations were previously determined to be suitable for detecting gut microbiota response to carbohydrates using these activity markers.^20^ Lactulose degradation by the gut microbiota was detected in all donor samples (Figure 1(a)). 29–58% of added lactulose was degraded by 6 h, indicating that, despite some interindividual variability in degradation kinetics, the gut microbiota from all donors demonstrated a considerable capacity to degrade lactulose.

### Lactulose drives both conserved and individualized microbial population dynamics

The gut microbiota of all donors was dominated on average by the phyla *Bacillota* (46.6%) and *Bacteroidota* (44.9%), with contributions from *Pseudomonadota* (4.6%) and *Actinomycetota* (3.8%; Figure 1(b)). Analysis of genus-level microbiome profiles of all samples revealed that most of the variation in the dataset was attributable to inter-individual differences, with donor-specific effects accounting for 78.6% of the total variation (R^2^ = 0.786, *p* = 0.001, *n* = 36 samples). This was also the primary driver of clustering observed in the principal coordinates analysis (PerMANOVA, *p* = 0.125; Figure 1(c)), reflecting considerable baseline differences in microbial composition across donors.

We next evaluated which bacterial genera grew upon lactulose amendment, as inferred by a significant increase in relative abundance, either in individual donor microbiomes or across the donor cohort. *Bifidobacterium* was the only genus that consistently increased in relative abundance across all six donors by 6 h in lactulose supplemented samples, but not in no amendment samples (DESeq2, Wald Test p.adj <0.05; Figure 1(d), Supplementary Figure S2). This observation aligns with prior studies reporting lactulose-induced bifidogenic effects in both in vitro and clinical settings.^19,21^ In addition, the relative abundances of *Collinsella, Gemmiger, Ruminococcus, Oscillibacter, Agathobacter, Anaerobutyrium*, *Escherichia*, and *Blautia* were significantly increased by 6 h in one or more donors, potentially reflecting a donor-specific response to lactulose supplementation (Supplementary Data 1). Incubations were also conducted with the two constituent monosaccharides of lactulose, galactose and fructose, to assess any differences in microbial responses and community shifts. *Bifidobacterium* was the most abundant genus that consistently increased in relative abundance in the presence of either sugar, though several other taxa were increased in one or both sugar in samples from one or more donors (Supplementary Figure S3, Supplementary Data 2–3).

### Lactulose induces translational activity in a subset of the microbiota

**Figure 2.**
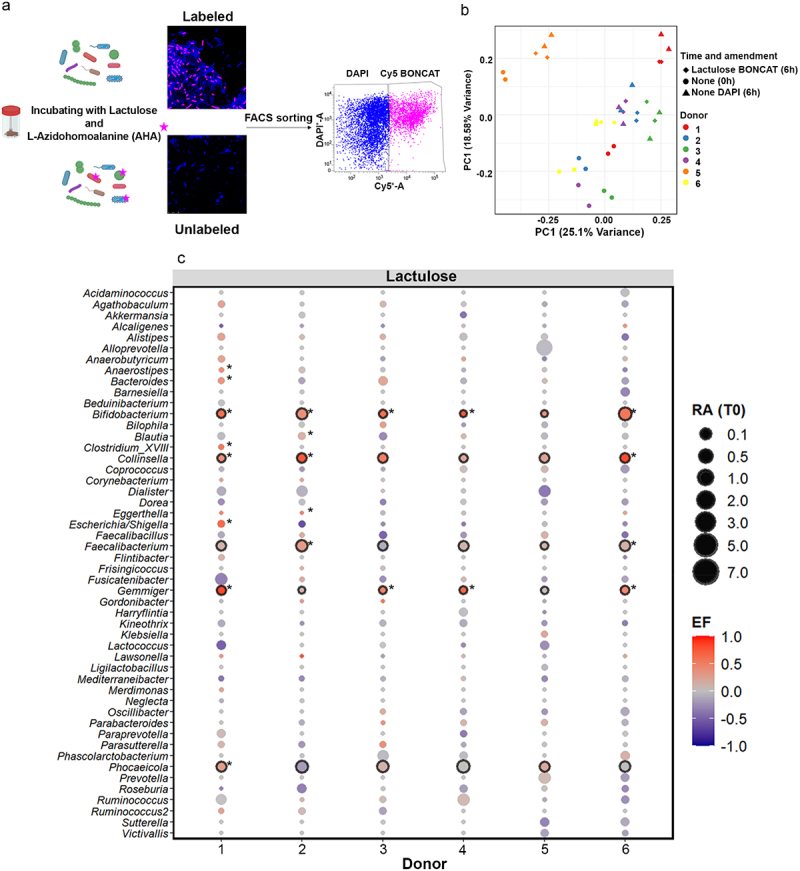
Identification of translationally-active cells. (a) Schematic representation of the workflow for sorting of translationally active cells using L-azidohomoalanine (AHA) labeling as a marker of cellular translational activity and bioorthogonal non-canonical amino acid tagging (BONCAT) with fluorescence-activated cell sorting (FACS).^20,23^ translationally active cells were visualized through Cy5 fluorescence (pink) in microscopy images, while total bacterial cells were stained with DAPI (blue). BONCAT-positive cells were then sorted using fluorescence-activated cell sorting (FACS) based on Cy5 fluorescence intensity (boncat, pink) and DAPI signals (blue). Sorted translationally active fractions represent metabolically active bacteria responding to lactulose, enabling downstream taxonomic identification via 16S rRNA gene sequencing. (b) Principal coordinate analysis (PCoA) ordination of genus-level microbiome profiles. “None (0h)” represents baseline (pre-incubation) microbiomes. “None DAPI (6h)” represents BONCAT-negative FACS-sorted cells after 6 h incubation of no amendment samples. “Lactulose BONCAT (6h)” represents BONCAT-positive FACS-sorted cells after 6 h incubation of lactulose-amended samples. Technical replicates were performed. (c) Change in relative abundance of abundant bacterial genera after 6 h incubation of lactulose-amended samples. Bubble size indicates relative abundance (RA) at 0h. The difference in relative abundance of each genus between BONCAT-positive FACS-sorted cells (lactulose BONCAT 6h) of lactulose-amended samples and BONCAT-negative FACS-sorted cells of no amendment samples after 6 h incubation (none DAPI 6h) was calculated as a normalized and scaled enrichment factor (EF; see materials and methods) and is indicated by bubble color. Genera significantly enriched in individual donors are marked with an asterisk and those significantly enriched across all six donors (as determined with DESeq2) are outlined in black.

As increases in relative abundance may not necessarily be a sensitive indicator of cellular response to lactulose, we next evaluated microbial stimulation by lactulose using L-azidohomoalanine (AHA) labeling as a marker of cellular translational activity, followed by bioorthogonal non-canonical amino acid tagging (BONCAT) with fluorescence-activated cell sorting (FACS).^20,22^ This approach allows for sorting and sequencing of translationally active as well as non-active cells (Figure 2(a)). Translationally active cells were detected by fluorescence microscopy in all lactulose-amended samples but not in no amendment samples after 6 hours of incubation, and therefore BONCAT-positive cells from 6 h lactulose incubations as well as BONCAT-negative cells from 6 h no amendment samples were sorted with FACS and subjected to 16S rRNA gene amplicon sequencing. Principal coordinates analysis (PCoA) of the genus-level microbiome profiles revealed donor-driven clustering (Figure 2(b); R^2^ = 0.5069, *p* = 0.001, *n* = 36 samples). However, BONCAT-positive cells from lactulose-amended samples clustered separately from non-active cells of no amendment samples from each donor (R^2^ = 0.1003, *p* = 0.001, *n* = 24 samples).

The genera that were most consistently stimulated by lactulose amendment across the donor cohort were *Bifidobacterium, Collinsella, Gemmiger, and Phocaeicola* (Figure 2(c), Supplementary Data 4). Multiple taxa were significantly stimulated in individual donors, including *Blautia, Clostridium, Eggerthella, Bacteroides, Faecalibacterium, Anaerostipes*, and *Escherichia/Shigella*. *Collinsella*, *Eggerthella, Faecalibacterium* and *Gemmiger* were also stimulated across all donors for both fructose and galactose incubations, potentially indicating their ability to metabolize these substrates (Supplementary Data 5–6). *Bacteroides* was stimulated across all donors in fructose incubations and *Bifidobacterium* was stimulated across all donors in galactose incubations (Supplementary Figure S4).

### Targeted cultivation of lactulose-stimulated bacteria by Raman activated cell sorting

**Figure 3.**
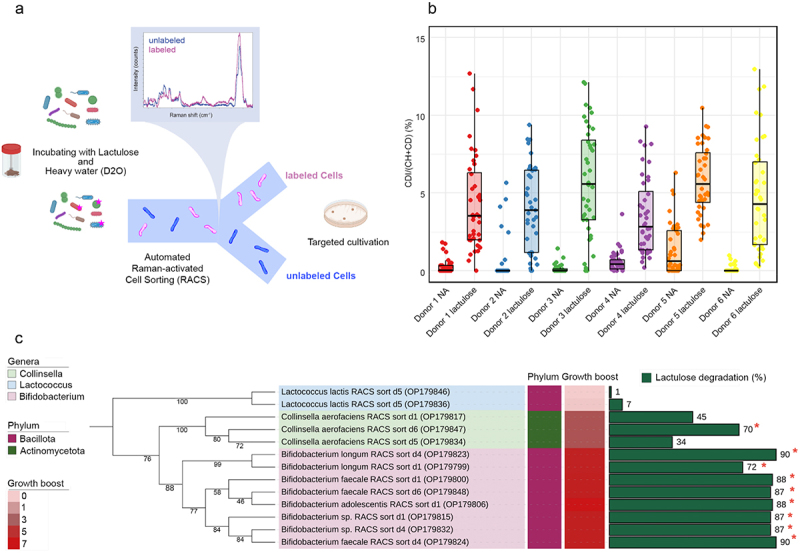
Sorting and physiological analysis of active cells. (a) Schematic of the detection and sorting of metabolically active cells via and D₂O labeling and Raman-activated cell sorting. Labeled cells (pink) exhibit a C-D peak at 2040–2300 cm^− 1^, indicating deuterium incorporation and metabolic activity, while unlabeled cells (blue) lack this signal. Targeted cultivation is performed after sorting the labeled cells. (b) Single-cell metabolic activity of cells. Dot plot showing the percentage of deuterium incorporation (%CD) into single microbial cells from fecal samples of 6 donors (D1–D6). Cells were incubated for 6 hours in D₂O-containing media with lactulose or without (no amendment, NA). Each dot represents a single-cell measurement, and boxplots summarize the distribution of %CD values. Statistical analysis across all donors confirmed a significant effect of lactulose treatment on microbial metabolic activity (ANOVA, *p* < 0.001, *n* = 471). (c) Identification and physiological characterization of lactulose stimulated cells. Phylogenetic tree of bacteria isolated using Raman-activated cell sorting. The phylogenetic tree was constructed using the maximum likelihood algorithm in IQ-TREE and rooted at the mid-point, with branch support evaluated using 1000 ultrafast bootstrap replicates. The aligned sequences were used to generate the tree, which was then visualized and annotated in iTOL. Several strains, including *Bifidobacterium sp., Bifidobacterium longum subsp. suillum*, and *Bifidobacterium faecale* showed ambiguous species-level assignments based on BLAST analysis. These strains matched multiple closely related species at ≥97% similarity, with some aligning to different species within the same genus. To maintain consistency in taxonomic classification, these isolates were assigned species names based on their highest-confidence BLAST match, acknowledging the limitations of 16S rRNA gene sequencing for precise species differentiation. This approach aligns with the conservative cut-off threshold described in the methods section, which included a 97% similarity threshold and the exclusion of uncultured sequences. Growth boost indicates the area under the growth curve in YCFA-lactulose relative to the no-amendment control. Lactulose degradation (%) shows the percentage of lactulose degraded after 24 hours of incubation. Asterisks (*) indicate strains with significant lactulose degradation (mean ± SD: 67.7% ± 31.8, *n* = 28) measured with HPAEC.

We next employed heavy water (D_2_O) activity labeling combined with Raman activated cell sorting (RACS)^20,24^ to isolate and physiologically characterize cells stimulated by lactulose amendment. The metabolic activity of individual microbial cells from incubations was assessed using Raman microspectroscopy, which measures the incorporation of deuterium (D) from D₂O into cellular structures, resulting in the formation of detectable C-D bonds (quantified as %CD; Figure 3(a)). Across all donors, %CD values were significantly higher in cells from lactulose-amended samples compared to no amendment samples, indicating stimulation of metabolic activity by lactulose (ANOVA, *p* < 0.001; Figure 3(b)).

Deuterium-labeled cells were then sorted from lactulose-amended samples using RACS and isolated by plating. Sorting and cultivation resulted in the recovery of 30 bacterial colonies, which were classified via near-full-length 16S rRNA gene sequencing. The isolates included *Collinsella* (*n* = 17), *Lactococcus* (*n* = 3), and *Bifidobacterium* (*n* = 10). This included several well-defined species such as *Bifidobacterium adolescentis, Bifidobacterium longum, Collinsella aerofaciens* and *Lactococcus lactis*, as well as strains with sequences that matched multiple closely related species within the same genus, reflecting a conservative genus-level classification where precise species identification was not possible (Figure 3(c), Supplementary Data 7). For comparison, direct isolation of bacteria on the same cultivation medium led to the isolation of species of *Bifidobacterium, Collinsella*, and *Phoecaicola* but not *Lactococcus* (Supplementary Figure S5), indicating a partial but incomplete overlap in recovered taxa with the two approaches.

One isolate of each species from each donor was then selected as a representative for physiological analysis. The addition of lactulose to cultivation medium stimulated the growth of all tested strains except *L. lactis* (Figure 3(c)). Likewise, significant lactulose degradation was observed by cultures of all tested *Bifidobacterium* spp. and some *C. aerofaciens* but not by *L. lactis*. For comparison, we conducted a random sorting experiment with RACS, in which cells were collected regardless of their metabolic activity, and isolated 20 strains. Only 7 of these strains were able to grow on a medium where lactulose was the main carbon source, underscoring the selectivity of the RACS approach (Supplementary Figure S6).

To gain insights into the enzymes responsible for lactulose degradation, we performed RNAseq on *B. adolescentis* grown in a medium containing either lactulose, lactose, or glucose. Among the six annotated β-galactosidase genes in its genome, one gene, CCMPN/A_D1907, was highly expressed in the presence of lactulose (Supplementary Figure 7). Interestingly, it was also highly expressed in the presence of lactose and has 83% sequence identity to the well-characterized *Escherichia coli* β-galactosidase responsible for lactose cleavage (LacZ, UniProt accession P00722). To confirm that a bacterial β-galactosidase can cleave both lactose and lactulose, we evaluated the enzymatic activity of *E. coli* β-galactosidase. Degradation of both lactulose and lactose, as well as release of their constituent monosaccharides, was observed under incubation conditions, albeit with slightly reduced activity in cleaving lactulose (Supplementary Figure 8).

### *Lactulose degraders support the growth of* L. lactis

**Figure 4.**
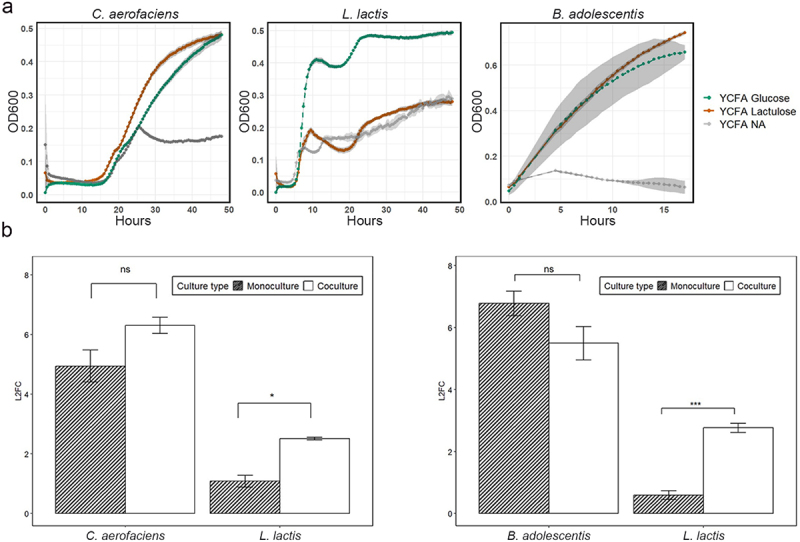
Pure and co-culture of strains in lactulose-containing medium. (a) Growth curves (OD600) for *L. lactis*, *C. aerofaciens*, and *B. adolescentis* were monitored in YCFA glucose, YCFA lactulose, and YCFA No amendment (NA) media. The growth curves of *L. lactis* and *C. aerofaciens* were measured over 48 hours, while *B. adolescentis* was measured over 15 hours reaching log phase. (b) Log22 Fold change in growth of *L. lactis* in lactulose-supplemented media was compared between monoculture and coculture conditions with *C. aerofaciens* and *B. adolescentis*. Significant growth enhancement of *L. lactis* (Student’s t-test, *p* < 0.05, *n* = 6) in coculture with *C. aerofaciens* and (Student’s t-test, *p* < 0.001, *n* = 6) in coculture with *B. adolescentis* is indicated with asterisks (*).

As *L. lactis* was labeled with heavy water but was unable to utilize lactulose, this raised the question as to whether growth of *L. lactis* was supported indirectly by lactulose degraders. To test this hypothesis, co-cultivation of *L. lactis* with lactulose degraders (either *C. aerofaciens* or *B. adolescentis*) was performed to evaluate whether inter-species interactions could enhance the growth of *L. lactis* in the presence of lactulose. As expected, *L. lactis* was unable to grow alone in a lactulose-supplemented medium while both *B. adolescentis* and *C. aerofaciens* grew well, reaching stationary phase in approximately 15 h and 48 h, respectively (Figure 4(a)). In co-culture in lactulose-supplemented medium, both *B. adolescentis* and *C. aerofaciens* facilitated the growth of *L. lactis*, as quantified by qPCR (Figure 4(b)). Notably, the abundances of *B. adolescentis* and *C. aerofaciens* were not significantly affected, positively or negatively, by the presence of *L. lactis*.

## Discussion

There is considerable interest in evaluating the potential for dietary supplements to promote a gut microbiota that supports health by, for example, enhancing digestion, training the immune system, protecting against the invasion of enteric pathogens, or producing desirable bioactive compounds such as SCFAs.^25–27^ One of the targets of prebiotics has been to promote the abundance of *Bifidobacteria*, which can provide numerous services in the gut.^28^ Previous studies have indicated that non-digestible polysaccharides such as inulin, FOS, and GOS may be promising bifidogenic dietary supplements that promote the growth of *Bifidobacterium* species in the gut, although not all studies are in agreement.^29–32^ Lactulose, a synthetic disaccharide composed of galactose and fructose, has been traditionally used as a laxative and in the treatment of hepatic encephalopathy, but it also exhibits prebiotic effects at low doses.^15^ This makes it a particularly valuable compound for investigating mechanisms of gut microbiota modulation in both healthy and disease contexts.^19^ Indeed, previous studies have reported increased *Bifidobacterium* abundance following lactulose supplementation not only in healthy adults but also in patients with liver disease, supporting its broader relevance as a microbiota-directed intervention.^19^ Lactulose is currently used primarily for clinical purposes and is not commonly consumed as a dietary supplement. However, our findings suggest that lactulose can selectively modulate gut microbes and promote cooperative interactions within the microbiota. These results underscore the need for further human studies to evaluate its potential as a microbiome-supporting and health-promoting dietary component. In this study, we employed multimodal cell sorting approaches to identify and characterize bacteria in the gut microbiota responsive to lactulose supplementation. Our findings demonstrate that *Bifidobacterium* spp., along with a few other taxa such as *Collinsella* and *Lactococcus*, are stimulated by the presence of lactulose. This is in line with the results of studies in humans that have evaluated lactulose’s bifidogenic effects.^19,21^ While previous studies have primarily linked lactulose fermentation to the growth of *Bifidobacterium* and *Lactobacillus* species,^13^ our findings reveal that lactulose also promotes *Collinsella* and *Lactococcus*, highlighting its broader but still targeted impact on gut microbiota composition. In addition, we observed translational activity in several other taxa, including *Gemmiger*, *Phocaeicola* and *Faecalibacterium*, suggesting that lactulose activates more microbial groups than previously recognized. This wider pattern of activation indicates that lactulose can enhance the metabolic activity of gut bacteria in ways that may not be visible through methods focused only on changes in abundance. Notably, taxa such as *Faecalibacterium* and *Gemmiger* which are generally considered beneficial for host health,^33–35^ showed metabolic activity in response to lactulose, highlighting its ability to selectively stimulate functionally important members of the gut microbiota. Lactulose is produced industrially by the isomerization of lactose.^36,37^ Although the biochemistry of bacterial lactose degradation is well characterized, bacterial lactulose degradation is less well studied. We identified a β-galactosidase gene (CCMPN/A_D1907) that *B. adolescentis* highly upregulates when grown on both lactose and lactulose. This enzyme shares homology with LacZ in *E. coli*, which cleaves lactose.^38^ We determined that the *E. coli* LacZ is also capable of cleaving lactulose, suggesting that LacZ homologs in other species may also have this dual activity. Future work should explore to what extent lactulose affinities of β-galactosidase variants encoded by differing members of the gut microbiota determine competitiveness in the lactulose utilization niche.

In addition to direct lactulose degradation, our study highlights the importance of cross-feeding interactions within the gut microbiota. The gut microbiota is an interconnected community in which microbes exchange metabolic products such as SCFAs and amino acids.^39,40^ We found that lactulose not only stimulates lactulose-degrading bacteria such as *B. adolescentis* and *C. aerofaciens* but also facilitates the growth of other species that do not directly degrade lactulose. This study demonstrates that *L. lactis*, a bacterium known for breaking down lactose in dairy products,^41^ becomes metabolically active in response to lactulose. We show that *L. lactis* is indirectly stimulated by primary degraders, despite its inability to directly cleave lactulose. Co-culture experiments confirmed that *B. adolescentis* and *C. aerofaciens* support the growth of *L. lactis*, likely via metabolite exchange. This finding reveals that *L. lactis* can benefit from interactions with lactulose-degrading bacteria, highlighting how lactulose can shape microbial communities through indirect metabolic support. Similar cross-feeding mechanisms have been described for other prebiotics such as inulin and FOS,^20,42^ where primary degraders release metabolites that benefit non-degraders, suggesting that lactulose may drive comparable ecological interactions. Our integrative approach of combining BONCAT-FACS, Raman-activated cell sorting (RACS), and transcriptomics allows for the identification of both primary degraders and metabolically stimulated non-degraders. This single-cell framework provides a mechanistic view of how prebiotics reshape community function, offering a platform for the functional evaluation of other microbiota targeted interventions. Our approach represents an expansion of previous single-cell activity-based approaches, as we not only identified primary lactulose degraders, but also evaluated downstream ecological interactions through co-culture experiments. This integrative strategy allowed us to identify specific ecological interactions that may otherwise remain undetected through compositional or activity-based analyses alone. This approach is broadly adaptable and could be extended to study other candidate prebiotics, ultimately contributing to a deeper understanding of microbial nutrient niches and inter-species interaction networks. One limitation of our study is that fecal samples were vortexed for 2–3 minutes during homogenization, which may have introduced mild mechanical stress. While this approach is consistent with widely used protocols in microbiome research and microbial activity was confirmed via BONCAT, Raman, and cultivation assays, we cannot fully exclude the possibility that this may bias recovery of fragile cell types. Future studies should consider testing alternative homogenization strategies to further minimize this potential bias. Further work is needed to determine whether monosaccharides produced from lactulose cleavage or other metabolic products are responsible for the facilitation of *L. lactis* growth, but these findings underscore the importance of microbial interactions and metabolic exchanges in shaping gut microbiota composition and function.

## Conclusions

This study provides new insights into the prebiotic potential of lactulose, highlighting its bifidogenic effects and broader impact on gut microbiota. By employing the advanced single cell techniques BONCAT-FACS and RACS, we identified lactulose-responsive bacteria in the gut microbiota. Our findings demonstrate that lactulose not only promotes the growth of *Bifidobacterium* but also enhances the translational activity of multiple other taxa such as *Collinsella*, *Gemmiger*, *Faecalibacterium* and *Phocaeicola*. Our study also highlights the importance of cross-feeding interactions between primary degraders and other species. These results highlight lactulose’s ability to modulate microbial activity and inter-species interactions, offering a mechanistic understanding of how gut communities respond to specific substrates. Unlike previous prebiotic studies that mainly rely on taxonomic shifts to infer microbial function, our approach demonstrates that lactulose directly activates metabolically important taxa and promotes cooperative interactions in the microbiome. Future work should further elucidate the inter-species interactions and ecological dependencies through which primary degraders of prebiotics support the growth of other community members and assess whether these microbial responses translate into health-related outcomes or justify the use of lactulose in prebiotic therapy.

## Materials and methods

### Fecal sample collection and anaerobic incubations

Fresh fecal samples were collected from 6 healthy participants (3 females and 3 males; BMI average: 23.3 ± 5.0). Participants who had taken antibiotics or consumed probiotic/prebiotic supplements within the past six months, or those with any chronic or acute intestinal conditions, were excluded from the study.^43,44^ Each participant collected their own sample using a sterile feces tube with a spoon and screw cap (Sarstedt, Germany). Health status was further assessed using a standardized questionnaire addressing diet, medication use, gastrointestinal history, and lifestyle factors. For the purposes of this study, participants were considered healthy if they reported no chronic or acute intestinal conditions and had not recently used antibiotics, probiotics, or prebiotics. The protocol was approved by the ethics committee of the University of Vienna, with all participating subjects signing written informed consent (reference number 00161).

Immediately after collection, fresh fecal samples were transferred into an anaerobic tent containing a gas mixture of 85% N₂, 10% CO₂, and 5% H₂.^45,46^ All subsequent handling steps, including mixing, vortexing, and filtration, were performed entirely within the anaerobic chamber to ensure that the microbial cells remained viable under oxygen-free conditions. To investigate microbial responses, lactulose, fructose, and galactose (2 mg/ml each, Carl Roth and Sigma-Aldrich)^47^ were used as substrates. A control set without any amendments was used to determine the baseline microbial activity in the absence of the added sugars. Approximately 1 g of fecal material was mixed with 10 ml of 2× PBS and vortexed vigorously for 2–3 minutes until homogenized, and then filtered through a 40 μm mesh (Corning, Germany) to eliminate large particulates. The filtrate was diluted 1:10 with 2x PBS in sterile tubes. 2 ml of each substrate was dissolved in D_2_O to autoclaved Hungate tubes containing 2 ml of the homogenized fecal material and 5 mM L-azidohomoalanine (AHA; Baseclick, Germany).^20^ The total volume was adjusted to 4 ml with final concentrations of 50 μM AHA and 50% D_2_O. Incubation was performed at 37 °C for 6 hours under anaerobic conditions. After incubation, the samples were washed with PBS to extract any remaining D_2_O and AHA. For preservation, 1 ml of each sample was fixed with 50% ethanol/PBS mixture and stored at −20 °C, and 1 ml was stored at −80 °C. for nucleic acids and metabolites analysis. For subsequent RACS experiments, samples were maintained in 20% glycerol/PBS and kept at −80 °C in crimp-sealed vials.

### BONCAT labeling and microscopy

In this experiment, lactulose was provided as a substrate to stimulate microbial activity, and AHA incorporation into newly synthesized proteins was used to directly identify translationally active cells.^22^ To visualize metabolically active microbial cells, fixed samples were prepared for fluorescence labeling on glass slides. Each fixed sample was pipetted 10 µl onto a microscopy slide (Marienfeld, Germany) and incubated at 46°C for 10 minutes to promote cell adhesion to the surface. The slides were then dehydrated by placing them for 3 minutes each in three ethanol solutions with increasing concentrations (50%, 80%, and 96%), and then allowed to air dry. For the labeling reaction, a dye premix was prepared by mixing 1.25 µl of 20 mM CuSO₄, 2.50 µl of 50 mM THPTA (tris(3-hydroxypropyltriazolylmethyl) amine), and 0.30 µl of Cy5 alkyne dye (Jena Bioscience, Germany). This mixture was kept in the dark at room temperature for 3 minutes to allow it to activate.^22^ In the next step, the dye premix was added to 221 µl of 1x PBS containing 12.5 µl of freshly prepared 100 mM sodium ascorbate and 12.5 µl of freshly prepared 100 mM aminoguanidine hydrochloride. The final mix was gently inverted once (without vortexing) to ensure proper mixing. From this final mixture, 30 µl were added to each well of the slide to cover the sample. The slides were then incubated in a humid chamber at room temperature for 30 minutes in the dark. During this time, the fluorescent dye reacted with the azide group of AHA (L-azidohomoalanine) in the cells, labeling metabolically active cells with a Cy5 signal. After the reaction, the slides were washed three times with 1x PBS for 3 minutes each. The dehydration step was repeated by placing the slides again into 50%, 80%, and 96% ethanol for 3 minutes each, followed by air drying. For DNA staining, 10 µl of DAPI (4′,6-diamidino-2-phenylindole) (Sigma-Aldrich, Austria) was added to each well, and the slides were incubated in the dark at room temperature for 10 minutes. Slides were briefly dipped in cold Milli-Q water, air-dried again, and mounted with glycerol. Finally, the slides were imaged using confocal laser scanning microscopy (CLSM). In the overlay images, DAPI-stained cells appeared blue, while metabolically active cells labeled with Cy5 appeared pink.^23^

### Liquid BONCAT and fluorescence-activated cell sorting

To identify metabolically active microbial cells, a BONCAT approach was employed in conjunction with FACS.^48,49^ The cells were labeled with a Cy5 alkyne dye via Cu(I)-catalyzed click chemistry, as previously described.^23^ In summary, the chemically fixed microbial cells were suspended in a click reaction mixture containing CuSO₄, THPTA, sodium ascorbate, aminoguanidine hydrochloride, and Cy5 alkyne dye.^22^ Approximately 300–500 μL of fixed sample was used per labeling reaction. Subsequently, the cells were washed and resuspended in PBS containing DAPI for the purpose of assessing total cell numbers.^50^ After labeling, Cells were gated based on DAPI fluorescence to detect DNA-containing cells and Cy5 fluorescence to identify BONCAT-positive cells. The full gating strategy, including controls and threshold definitions, is provided in the supplementary information (Supplementary Figure S9). The labeled cells were sorted according to their Cy5 fluorescence intensity using the cell sorter FACS Melody (BD, Germany). A total of 500,000 events were collected per sample at a flow rate of 10 μL/min.^51^

### 16S rRNA gene amplicon sequencing

To characterize the microbial community composition, DNA was extracted from both the entire fecal sample (0 and 6 hours incubation), the entire sorted microbial community stained with DAPI and the subpopulation of metabolically active cells (FACS-sorted cells) after 6 hours of incubation with lactulose, fructose, galactose, or no amendment. The QIAamp DNA Mini Kit (Qiagen, Germany) was utilized for DNA extraction, incorporating an additional lysozyme step to enhance recovery.^52,53^ The 16S rRNA gene was subsequently amplified using a dual-barcoding, two-step PCR two-step approach,^54^ to determine the microbial community structure. The V3–V4 hypervariable region was targeted using primers 341F (5′-CCT ACG GGN GGC WGC AG-3′) and 785 R (5′-GAC TAC HVG GGT ATC TAA TCC-3′), with 16-bp head adapters (H1: 5′-GCT ATG CGC GAG CTG C-3′; at the 5′ end of both the forward and reverse primer) added during the first PCR step.^55^ PCR products were purified and normalized using SequalPrep™ Normalization Plate Kit (Invitrogen), which also removes primer dimers and normalizes DNA yield followed by a second PCR step with unique dual barcodes.^54^ Samples were pooled and concentrated using the innuPREP PCRpure Kit (Analytik Jena) and sequencing libraries were prepared using the Illumina TruSeq Nano DNA Library Prep Kit. Libraries were sequenced in paired-end mode (2 × 300 bp, v3 chemistry) on an Illumina MiSeq platform at the Joint Microbiome Facility of the Medical University of Vienna and the University of Vienna (Project ID JMF-2102–07). Demultiplexed reads were processed using the DADA2 pipeline (v1.18.0) to infer amplicon sequence variants (ASVs).^56^ The pipeline included primer trimming, quality filtering, error modeling, dereplication, sample inference, merging of paired-end reads, chimera removal, and ASV table construction. Taxonomic assignment was performed using the SILVA SSU Ref NR 99 database (release 138.1).^57^ Contaminant sequences were filtered using the prevalence-based method from the decontam R package (v1.6.0).^58^ The resulting sequence data have been deposited in the NCBI Short Read Archive under accession number PRJNA718139.

### Raman microspectroscopy on slides

Fecal samples were amended with lactulose and incubated for 6 hours. An unamended control sample was included for comparison.^59^ Both lactulose-amended and control samples were fixed with ethanol:PBS (1:1) to preserve cellular integrity. For analysis, 1.5 μL of each fixed sample was spotted onto an aluminum-coated slide (Al136; EMF Corporation, USA) and air-dried at 30°C.^59^ Subsequently, the slides were dipped in ice-cold Milli-Q water (Millipore, Austria) twice to remove residual buffer components and air-dried again.^60^

Raman spectra of individual microbial cells were acquired using a confocal Raman microspectroscope (LabRAM HR800, Horiba Scientific, France) equipped with a 532 nm neodymium-yttrium aluminum garnet (Nd:YAG) laser and a 300 grooves/mm diffraction grating.^61^ Spectra were collected in the wavenumber range of 400–3200 cm^− 1^ using LabSpec 6 software (Horiba Scientific, France) with an acquisition time of 10 seconds and 5 accumulations per measurement. For each sample, 30–40 individual cells were analyzed.

The degree of deuterium substitution (%CD) in C-H bonds was quantified by integrating the peak areas of the C-D (2040–2300 cm^− 1^) and C-H (2800–3100 cm^− 1^) stretching vibrations using the single-cell analysis and testing tools for Raman microspectroscopy (Scattr) (https://shiny.lisc.univie.ac.at/scattr/).^62^ The threshold for %CD was determined by calculating the mean + 3 times the standard deviation (SD) of %CD values obtained from randomly selected cells in a control sample incubated without lactulose or D₂O addition.^62^ Raman data has been deposited in MicrobioRaman platform within the BioStudies database^63^ with accession numbers S-MBRS12.

### Raman-activated cell sorting

Raman-activated cell sorting (RACS) was performed on cells incubated for 6 hours in a medium containing D₂O and lactulose. After incubation, the cells were preserved in 20% glycerol balanced with PBS and stored at −80°C. Before sorting, the frozen cells were thawed, pelleted by centrifugation at approximately 9000×g for 7 minutes, washed twice with 0.3 M glycerol/MQ water buffer to minimize osmotic stress, and finally resuspended in 500 μl of the same solution.

The RACS platform combined a Raman microspectroscope operating at 532 nm and 90 mW, optical tweezers employing a 1064 nm Nd:YAG laser at 500 mW, and a polydimethylsiloxane (PDMS) microfluidic device.^24,64^ The microfluidic system was fabricated using a base and curing agent mixed at a 10:1 ratio, polymerized at 75°C, and mounted on a glass coverslip.^64^ This system featured two outlets designed to default the sample stream through the waste outlet unless redirected by optical tweezers.^64^

During sorting, cells were identified based on their Raman spectra, with deuterium-labeled cells being translocated to a collection outlet, while unlabeled cells were discarded through the waste outlet.^64^ The sorting process was automated using in-house MATLAB software (version 4.2), employing two indices: the cell index (Pc), indicating cell capture, and the labeling index (PL), differentiating labeled from unlabeled cells. Pc measured spectrum of Raman signal in the region between 1620–1670 cm^−1^ and the integrated intensity I (Pc = $\frac{I1620\;-1670\;}{I\;\mathrm{fluid}1620-1670\;}$).^24^ This specific region was chosen as it is largely unaffected by the Raman spectrum of used materials polydimethylsiloxane (PDMS) and glass. On the other hand, the ‘labelling index’ PL is determined by the comparison of the region of the deuterium peak in the Raman spectrum 2,040–2,300 cm^−1^ (integrated intensity: I2,040–2,300) to the region between 1,850–1,900 cm-1 (integrated intensity: I1,850–1,900) (P_L_ $=\frac{I2040-2300}{I1850-1900}$).^24^ The threshold for PL was set at 5.7 (*n* = 110 cells) based on random cell sorting amended with glucose and incubated without deuterated water(D2O).

After sorting, 50 μl of the sorted cells were collected, immediately transferred to an anaerobic tent, and cultured on YCFA. The cells were incubated at 37°C until colonies appeared, then streaked on new plates with YCFA-lactulose medium and cultured again to ensure colony formation. To verify the purity of the 0.3 M glycerol/MQ water buffer used during sorting, an aliquot of the buffer was cultured overnight under the same conditions. No microbial growth was observed, confirming it as a sterile negative control.^20,59^

### Taxonomic identification of bacterial isolates

For the molecular characterization of isolated bacterial colonies, colony PCR was performed to amplify the 16S rRNA gene. The PCR master mix was prepared under sterile conditions in the PCR hood and included 5 μL of 10x Green Dream Taq Buffer including 20 mM MgCl2 (Thermo Fisher Scientific, Waltham, MA, USA),5 μL of dNTPs 2 mM (Thermo Fisher Scientific),1 μL of each primer (forward: 616 V, 5’-AGA GTT TGA TYM TGG CTC AG-3’; reverse: 1492 R, 5’-GGT TAC CTT GTT ACG ACT *T*-3’) at a final concentration of 50 μM (Thermo Fisher Scientific),0.5 μL of BSA 20 mg/mL (Thermo Fisher Scientific), 0.5 μL of Dream Taq DNA Polymerase 5 U/μL (Thermo Fisher Scientific) and 37 μL of nuclease-free water (Thermo Fisher Scientific) in a final volume of 50 μl per reaction. The amplification protocol followed an initial denaturation at 95 °C for 3 minutes, then 30 cycles at 95°C for 30 seconds, 56°C for 30 seconds, 72°C for 1.5 minutes, and a final elongation at 72 °C for 10 minutes.^65^ Amplified PCR products were visualized via 1% agarose gels electrophoresis and purified using the InnuPREP PCRpure Kit (Analytik Jena, Germany) according to the manufacturer’s instructions.^66^ The concentration of each purified product was quantified using a Nanodrop device (Thermo Fisher Scientific, MA, USA).^67^ Subsequently, samples were sent to Microsynth AG (Vienna, Austria) for Sanger sequencing. Sequencing results were analyzed using a combination of 4Peaks 1.8 (Nucleobytes, Amsterdam, Netherlands) and Serial Cloner 2.6 for alignment, and the online tool DECIPHER to check for chimeras.^68^ To identify the bacterial taxa, the NCBI nucleotide collection database (https://blast.ncbi.nlm.nih.gov/) was employed. All sequences were compared to the NCBI database using a minimum threshold of 96% sequence similarity. Most isolates showed ≥ 98% identity to known reference strains, supporting reliable species-level assignments. However, for sequences with 96–97% similarity that matched multiple closely related species, a conservative genus-level classification was adopted to account for the limitations of 16S rRNA gene sequencing for precise species differentiation. This approach included the use of a 97% similarity cutoff for genus-level classification, as sequences within this range often align to multiple closely related species. Uncultured sequences were excluded to improve taxonomic accuracy and focus on well-characterized type strains, reducing the risk of misclassification. The sequences were then deposited in GenBank, with accession numbers OP179799-OP179820, OP179823, OP179824, OP179826-OP179849.

### Growth characterization of isolates

Strains were cultured in both solid and liquid YCFA medium until reaching the early stationary phase. Each strain, identified via Sanger sequencing, was grown in YCFA-glucose (YCFA-G) broth at 37°C in an anaerobic environment. Upon reaching maximum OD600, cultures were washed and diluted into YCFA broth supplemented with lactulose (YCFA-Lact) and YCFA media without any amendment (YCFA-NA), serving as a negative control. These cultures were dispensed into wells of a sterile 96-well microtiter plate (Costar 3595, Corning, NY, USA) in three technical replicates. The plate was incubated for 120 hours at 37°C in an anaerobic microplate reader (MultiskanTM GO, ThermoFisher Scientific) using SkanIt Software RE for Microplate Readers RE, ver. 6.1.0.51 (ThermoFisher Scientific) for shaking (5 seconds) and OD600 measurement every 30 minutes.^20^ Growth curves were subsequently calculated using R statistical software (https://www.r-project.org/). Supernatants from these cultures were collected and stored at −80°C for further HPAEC analysis.

### Lactulose degradation analysis by high-performance anion-exchange chromatography with pulsed amperometric detection (HPAEC-PAD)

Bacterial strains isolated RACS and fecal samples were analyzed for lactulose degradation by high performance anion exchange chromatography (HPAEC) utilizing a Dionex ICS 3000 system (ThermoFisher, Germany).^69^ Supernatants were centrifuged at 24,000 × g, and clear supernatants (20 μl) were diluted with 480 μl of 20% v/v methanol and filtered into injection vials using a syringe filter (0.22 μm, polyethersulfone membrane). The samples were injected into an ICS-3000 system (Thermo Fisher Scientific Inc., Sunnyvale CA, USA) . The sugars were separated on a Dionex CarboPac PA20 column (3 × 150 mm) with a Dionex CarboPac PA20 guard column (3 × 30 mm), using a solvent of 100 mM KOH, at a flow rate of 0.3 ml/min, over 35 minutes.^70^ Duplicate analyses were performed employing the standard carbohydrate waveform for detection. Calibration curves were established utilizing standards of fructose and galactose (Sigma-Aldrich, St. Louis, MO, USA), with lactulose (Carl Roth) employed for experimentation and measurement via HPAEC.

### Coculture experiments

To determine the optimal time points for late log-phase growth, growth curves were first established for *L. lactis, C. aerofaciens*, and *B. adolescentis* in YCFA medium supplemented with Lactulose, Glucose, or left without amendment (NA).^20^ Following this, each bacterial strain was grown as both monocultures and cocultures to assess their interactions. For coculture experiments, equal volumes (50 µL each) of log-phase cultures were mixed. Samples were collected at 0 hours, 15 hours, and 48 hours to monitor growth patterns and capture the late log-phase activity in both pure and mixed cultures.

Primer design was performed using Primer3Plus to generate strain-specific primers for *L. lactis, C. aerofaciens*, and *B. adolescentis* .^71,72^ The primers used were Coll16S-F (5´-TGCTACAATGGCCGGTACAG-3´) and Coll16S-R (5´-AGCAACTCCGACTTCATGGG-3´) for *C. aerofaciens* (product length: 114 bp, Tm: 68°C), Lact16S-F1 (5´-CTCTAACGAGACTGCCGGTG-3´) and Lact16S-R1 (5´-GCGACTCGTTGTACCATCCA-3´) for *L. lactis* (product length: 113 bp, Tm: 68°C), and Bifido16S-F (5´-TCCGGTGTGAAAGTCCATCG-3´) and Bifido16S-R (5´-CCGTTACACCGGGAATTCCA-3´) for *B. adolescentis* (product length: 94 bp, Tm: 68°C). Primer specificity was validated by performing PCR with each strain using its specific primer and primers designed for the other strains, followed by gel electrophoresis to verify the absence of nonspecific amplification products.^73^ Gradient PCR was utilized to optimize primer annealing temperatures for precise binding.^74^ DNA standards were prepared by culturing each strain to an optical density (OD) of 0.1 at 600 nm, followed by DNA extraction.^75–77^ To optimize qPCR reproducibility and allow reliable comparisons of DNA concentrations across samples, we established a calibration curve using 10-fold serial dilutions of DNA extracted from pure cultures. Subsequently, qPCR was performed using these strain-specific primers to quantify the abundance of *L. lactis, C. aerofaciens* and *B. adolescentis* in both pure cultures and cocultures at various time points. Each sample was tested in triplicate.

### RNAseq of Bifidobacterium adolescentis

*B. adolescentis* was revived from glycerol stocks by inoculating 50 µl of the pure culture into 5 mL YCFA-Glucose broth and incubated overnight at 37°C under anaerobic conditions. Cells were harvested by centrifugation at 13,000 rpm for 1 minute, washed twice with 500 µl 1× PBS, and resuspended in 1 mL 1× PBS. For the experimental setup, 50 µl of the washed cell suspension was inoculated into 10 mL of MRS medium containing one of the three substrates: glucose, lactose, or lactulose. Cultures were grown in four biological replicates under anaerobic conditions at 37°C until the late log growth phase. At the late log phase, cultures were centrifuged at maximum speed for 10 minutes at 4°C in 15 mL Falcon tubes. Supernatants were carefully removed, and the cell pellets were stored at −80°C for subsequent RNA extraction. Sequencing libraries were prepared from DNA-clean RNA extracted using the NEBNext Ultra II FS Directional Library Preparation Kit, after rRNA depletion with the RiboZero Plus Kit (Illumina), following the manufacturer’s instructions. Libraries were pooled equimolarly and sequenced on a NovaSeq6000 (Illumina) in paired-end mode (2 × 100 bp, 200 cycles) at the Joint Microbiome Facility (JMF) of the University of Vienna and Medical University of Vienna (Project ID: JMF-2212–19). Gene expression levels were quantified in fragments per kilobase of transcript per million mapped reads (FPKM).^78^ Differentially expressed genes were identified using DESeq2 with statistical significance determined at an adjusted p-value cutoff of 0.05.^79^ Heatmaps displaying log2 fold change (L2FC) in gene expression across substrate conditions were generated using the pheatmap package in Rstudio. The RNA sequencing data generated from this study are available in the NCBI Sequence Read Archive (SRA) under accession number [PRJNA1208440].

### Statistical analysis

Statistical analyses were performed using R statistical software (https://www.r-project.org/., v4.2.1).^80^ Data visualization was conducted using the ggplot2 package (v3.5.1).^81^ To analyze the Enrichment Factor (EF) between samples, the relative abundance (RA) of bacterial genera was calculated for lactulose-amended samples by comparing the differences between BONCAT-positive FACS-sorted cells and BONCAT-negative FACS-sorted cells. The EF was computed using the relative abundance of each genus at 6 hours in the lactulose-amended samples compared to its relative abundance at 0 hours. A positive EF (>0) indicates that a genus was enriched in the lactulose-amended samples, while a negative EF (<0) indicates that a genus was depleted relative to its baseline abundance at 0 hours.

For the differential abundance testing, the DESeq2 (v1.38.3)^82^ package was used to analyze the variation between groups (lactulose-amended vs. control). All tests were performed at a significance threshold of p-value <0.05, and multiple comparisons were corrected using the Benjamini-Hochberg method to control the false discovery rate (FDR).^83^ This correction method ensures that the risk of false positives is minimized when testing many hypotheses simultaneously. The resulting dataset contains the log2 fold changes (LFC) for each genus, along with the associated p-values and adjusted p-values (FDR). Genera with an adjusted p-value <0.05 were considered statistically significant and included in the final analysis. Bubble plots were used to visualize the results, where the bubble size represents the relative abundance at baseline (0 h), the color indicates whether the genus was significantly enriched or depleted in the lactulose-amended samples. and statistical significance was determined for each genus.

Additionally, PERMANOVA (Permutational Multivariate Analysis of Variance) was employed to evaluate community-wide differences in the microbiota profiles of lactulose-amended versus control samples. This was carried out using the vegan (v2.6.4) package, which is designed for multivariate statistical analysis.^84^ The adonis function from the vegan package was used for calculating significance of differences between groups, based on Bray-Curtis dissimilarity distances calculated from genus-level abundance data.^85^ This allowed for the testing of whether differences between sample groups were statistically significant in a multivariate context.

## Supplementary Material

Hamid_Lactulose_SI_130525.docx

## Data Availability

The 16S rRNA gene amplicon sequence data generated in this study from FACS have been deposited in the NCBI Short Read Archive under accession number PRJNA718139. The sequences of RACS strains isolated following lactulose supplementation have been deposited in GenBank, with accession numbers OP179799–OP179820, OP179823, OP179824, and OP179826–OP179849. Sequences from random sorting by RACS and isolation via direct sample plating on YCFA-Lactulose agar Plates are also available in GenBank, with accession numbers PQ722470–PQ722509. Raman data have been deposited in the MicrobioRaman platform within the BioStudies database under accession number S-MBRS12. RNA sequencing data generated from this study are available in the NCBI Sequence Read Archive (SRA) under accession number PRJNA1208440.
