# Supplementary material for: Lactulose selectively stimulates members of the gut microbiota, as determined by multi-modal activity-based sorting
Source: Gut Microbes. 2025 Jun 27;17(1):2525482. doi: 10.1080/19490976.2025.2525482 (PMC12218441; doi:10.1080/19490976.2025.2525482)
Supplement: Hamid_Lactulose_SI_130525.docx [file KGMI_A_2525482_SM3652.docx]

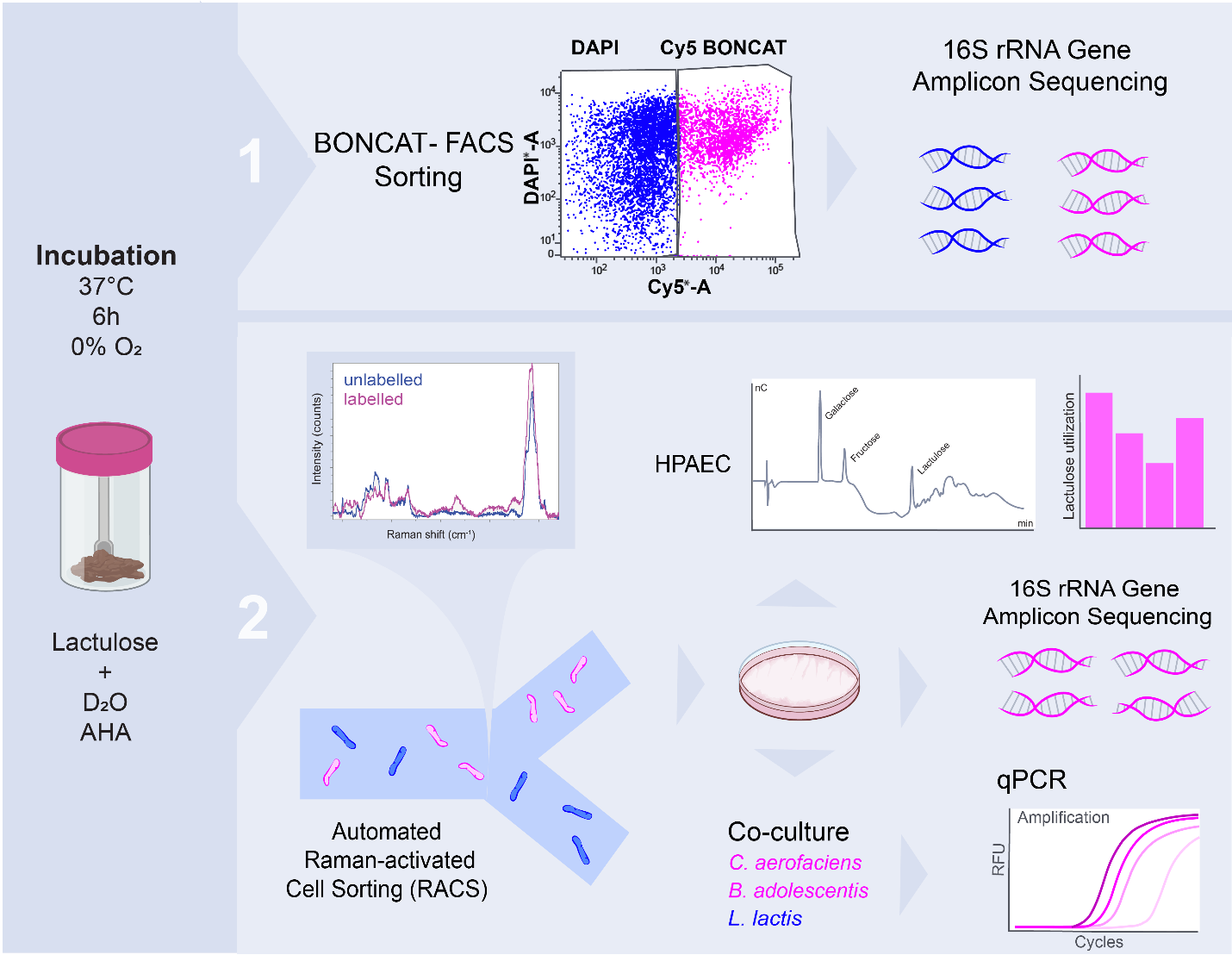


**Supplementary Figure 1. Outline of experiments and analyses.** Fresh fecal samples were anaerobically incubated with lactulose of its component monosaccharides as well as the cellular activity markers D₂O and L-azidohomoalanine (AHA). Bioorthogonal non-canonical amino acid tagging coupled with fluorescence-activated cell sorting (BONCAT-FACS) was used to detect metabolically active and inactive cells by 16S rRNA gene amplicon sequencing. Raman-activated cell sorting (RACS) was then employed for targeted isolation of active cells, which were subsequently identified by 16S rRNA gene amplicon sequencing and whose lactulose degradation capability was determined by high-performance anion-exchange chromatography (HPAEC). Coculture experiments with *Collinsella aerofaciens*, *Bifidobacterium adolescentis*, and *Lactococcus lactis* were conducted, and qPCR was used to monitor growth.


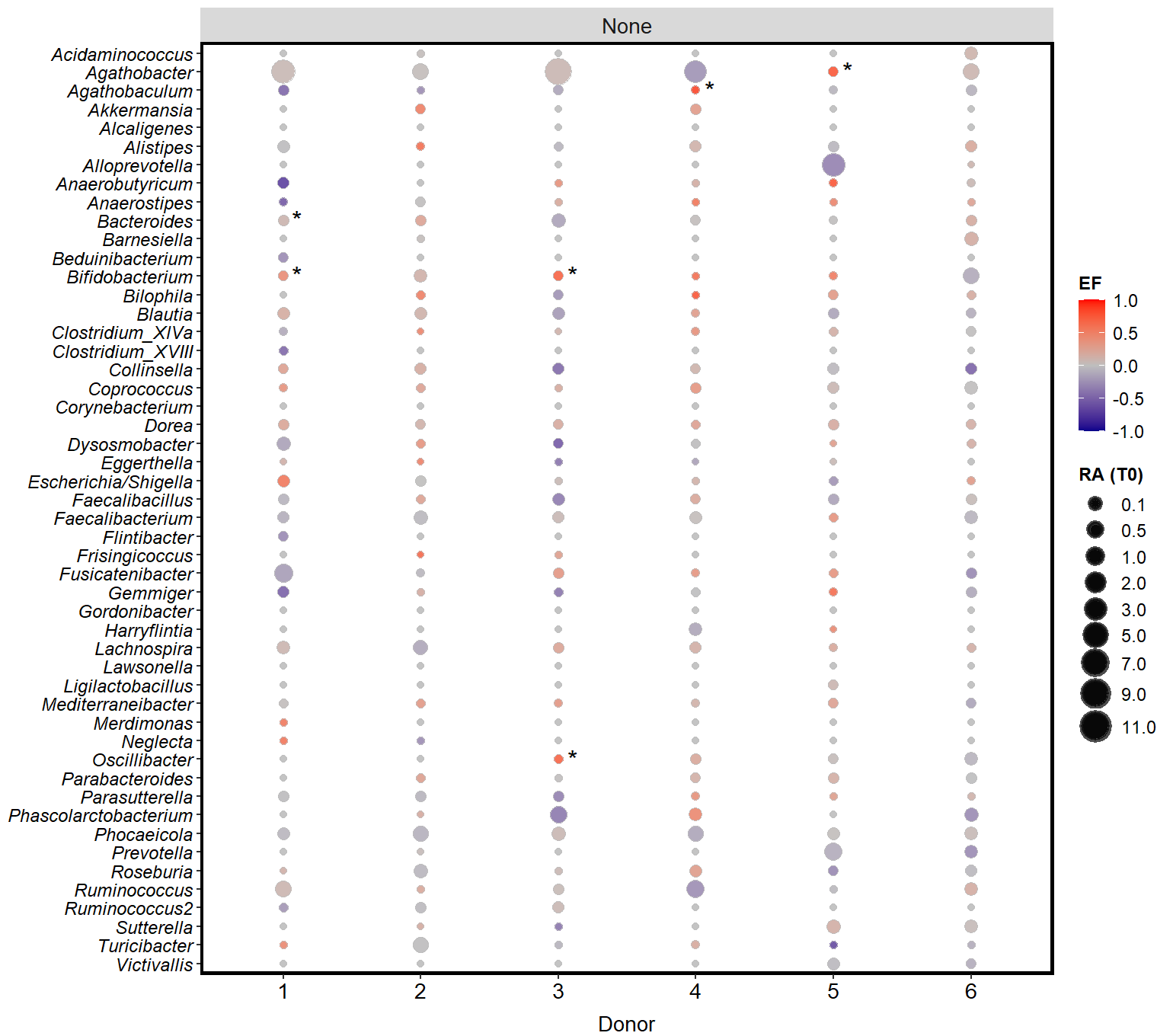


**Supplementary Figure 2. No amendment control.** Change in relative abundance of abundant bacterial genera after 6 h incubation in no amendment samples. Bubble size indicates relative abundance (RA) at 0h. The change in relative abundance during incubation, calculated as a normalized and scaled enrichment factor (EF), is indicated by bubble color. Genera significantly enriched in individual donors are marked with an asterisk and those significantly enriched across all six donors (as determined with DESeq2) are outlined in black.


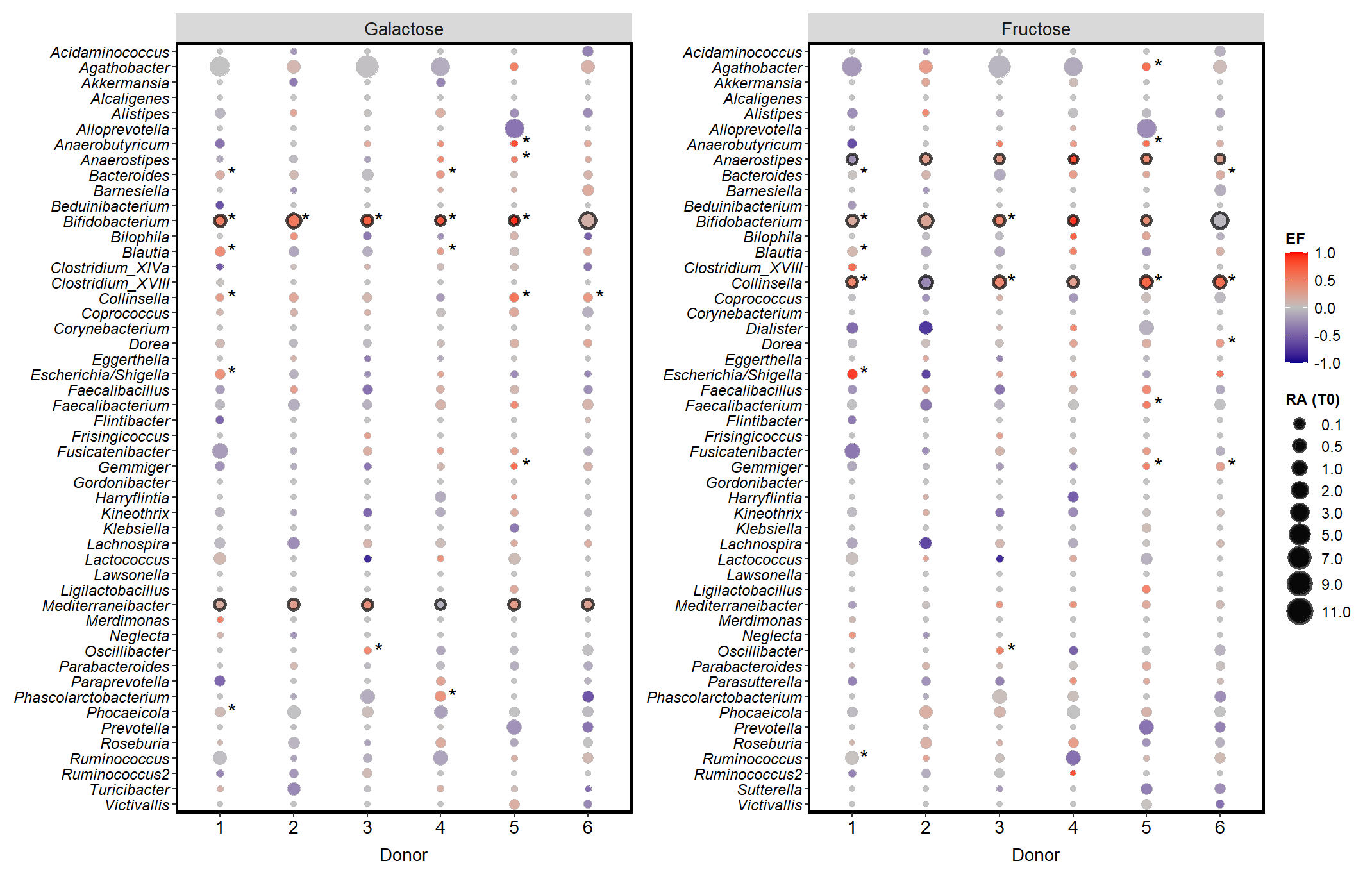


**Supplementary Figure 3. Incubation with galactose and fructose.** Change in relative abundance of abundant bacterial genera after 6 h incubation in galactose- and fructose-amended samples. Bubble size indicates relative abundance (RA) at 0h. The change in relative abundance during incubation, calculated as a normalized and scaled enrichment factor (EF), is indicated by bubble color. Genera significantly enriched in individual donors are marked with an asterisk and those significantly enriched across all six donors (as determined with DESeq2) are outlined in black.


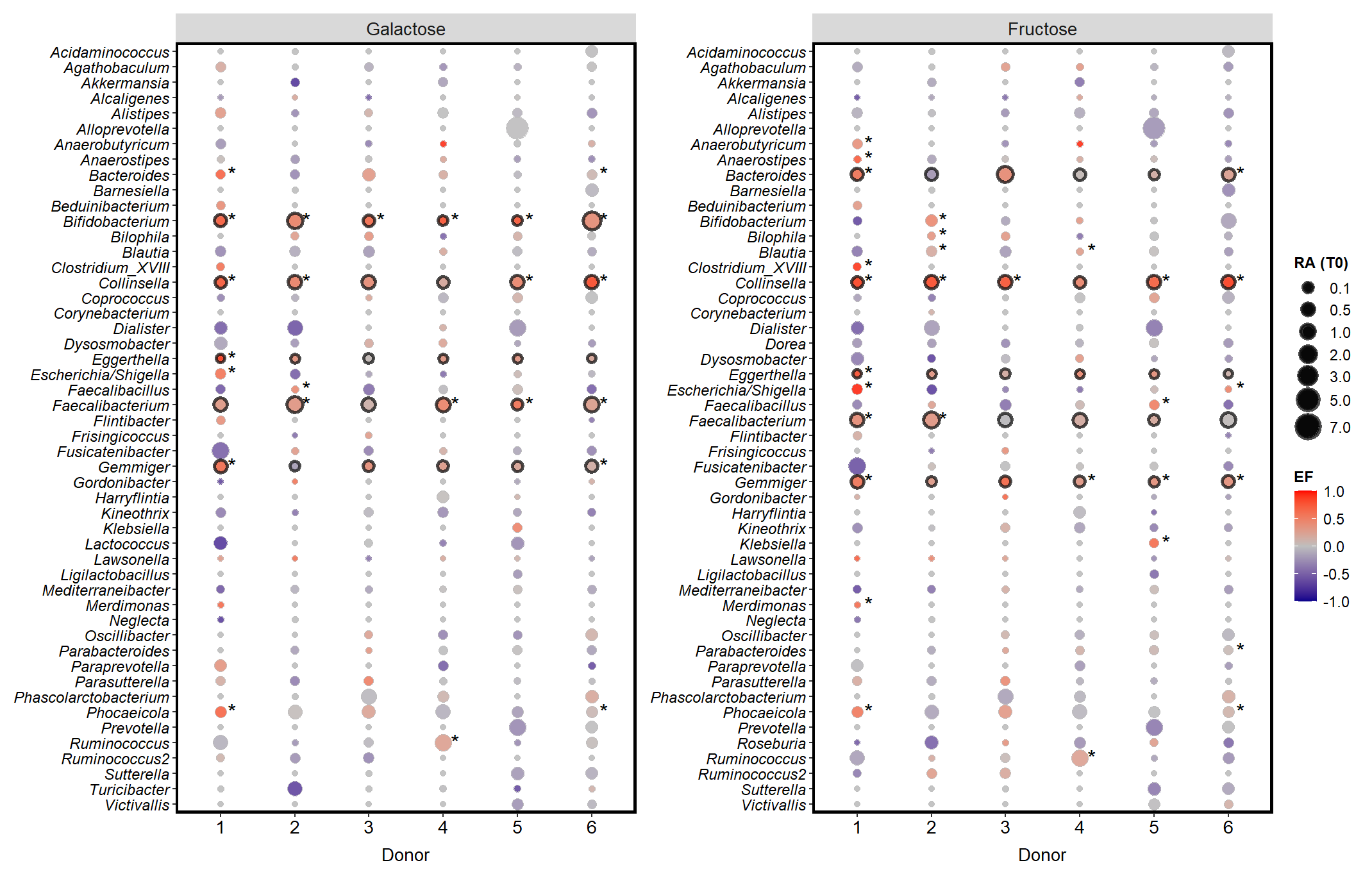


**Supplementary Figure 4. Identification of translationally-active cells.** Change in relative abundance of abundant bacterial genera after 6 h incubation in galactose- and fructose-amended samples. Bubble size indicates relative abundance (RA) at 0h. The difference in relative abundance of each genus between BONCAT-positive FACS-sorted cells of lactulose-amended samples and BONCAT-negative FACS-sorted cells of no amendment samples after 6 h incubation was calculated as a normalized and scaled enrichment factor (EF; see Materials and Methods) and is indicated by bubble color. Genera significantly enriched in individual donors are marked with an asterisk and those significantly enriched across all six donors (as determined with DESeq2) are outlined in black.


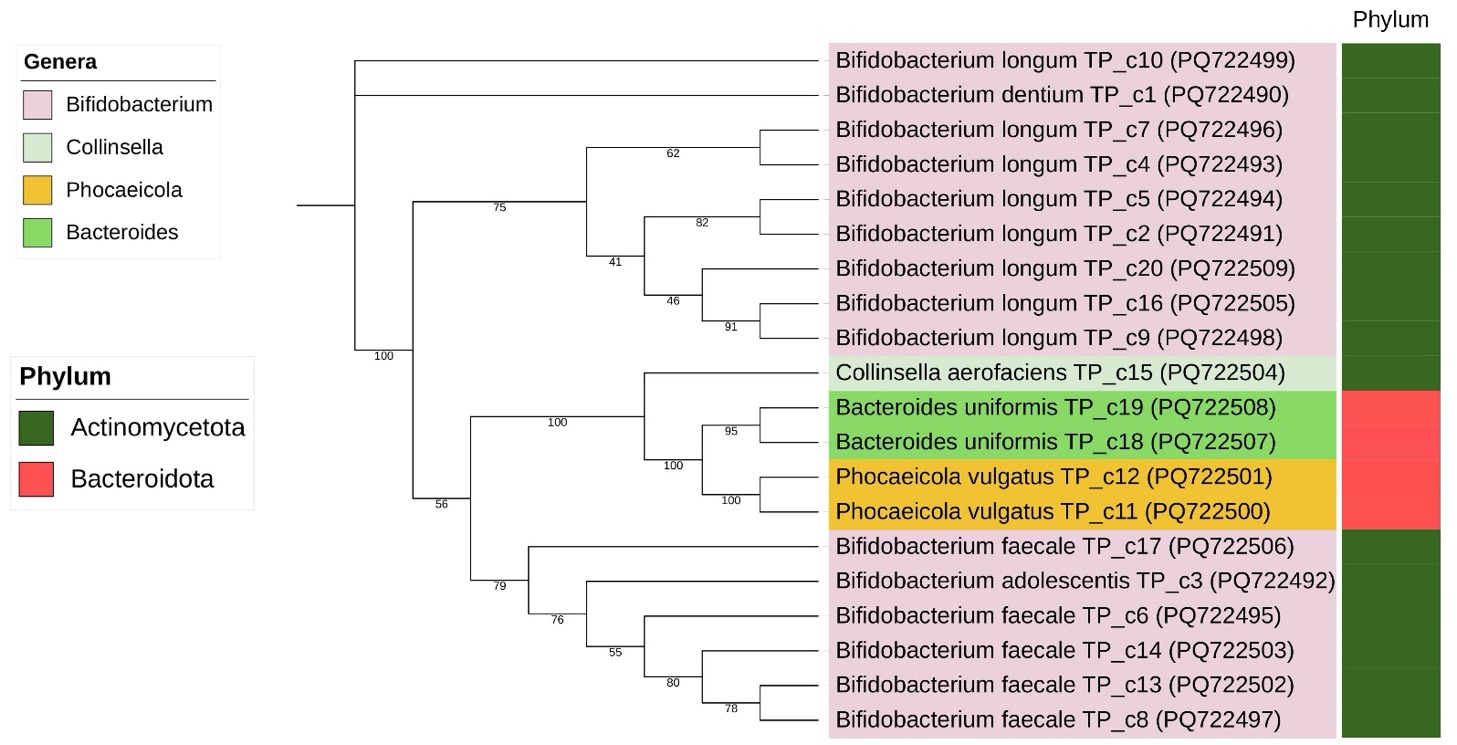


**Supplementary Figure 5. Isolation of Lactulose-Utilizing Bacteria Using Direct Plating on YCFA-Lactulose Agar Plates**. Phylogenetic tree showing the bacterial strains isolated from two fresh fecal samples grown on YCFA lactulose agar plates for 24 hours. The phylogenetic tree was constructed using the maximum likelihood algorithm in IQ-TREE and rooted at the mid-point, with branch support evaluated using 1000 ultrafast bootstrap replicates. The aligned sequences were used to generate the tree, which was then visualized and annotated in iTOL. Colonies were identified through colony PCR and Sanger sequencing.


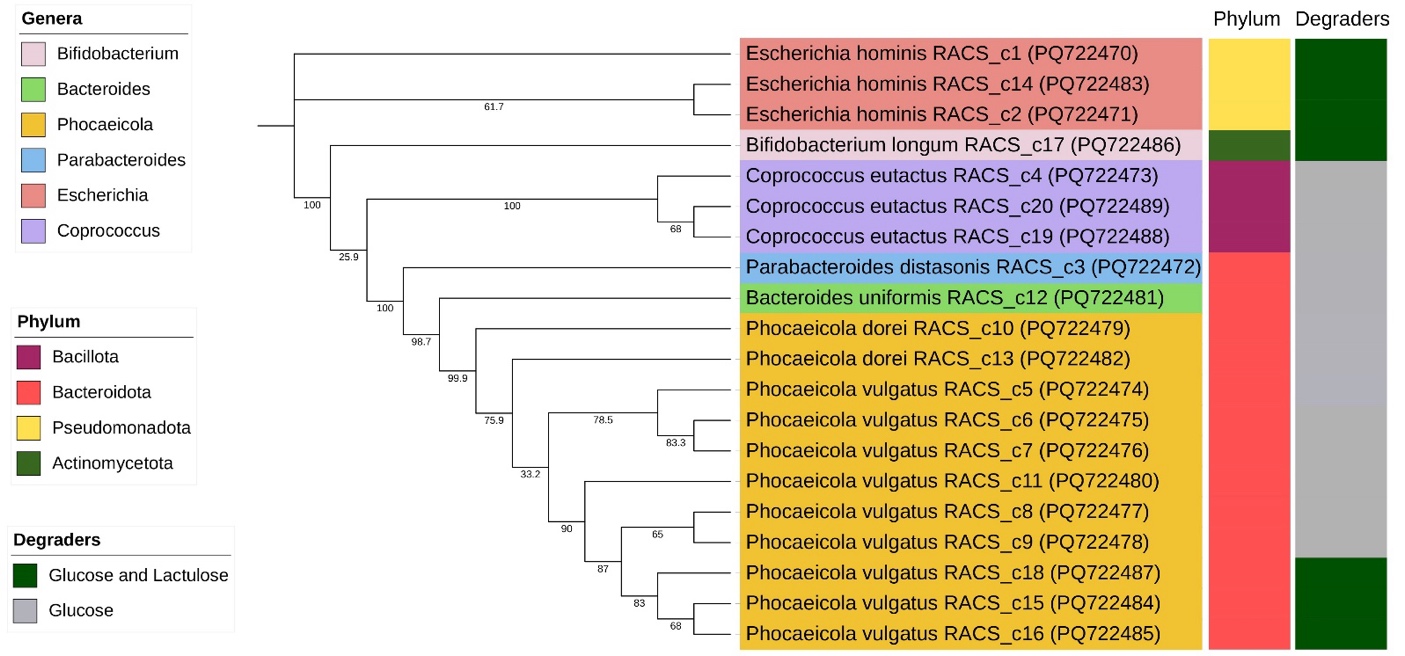


**Supplementary Figure 6. Random Sorting by RACS**. A random sorting experiment was used to capture all bacterial cells in the sample, regardless of their activity. Sorted cells were grown on YCFA Glucose agar plates and subsequently transferred to YCFA Lactulose agar to assess lactulose degradation. Green bars indicate strains capable of growing on both YCFA Glucose and YCFA Lactulose (lactulose degraders), while gray bars represent strains that failed to grow on lactulose. The phylogenetic tree was constructed using the maximum likelihood algorithm in IQ-TREE and rooted at the mid-point, with branch support evaluated using 1000 ultrafast bootstrap replicates. The aligned sequences were used to generate the tree, which was then visualized and annotated in iTOL.


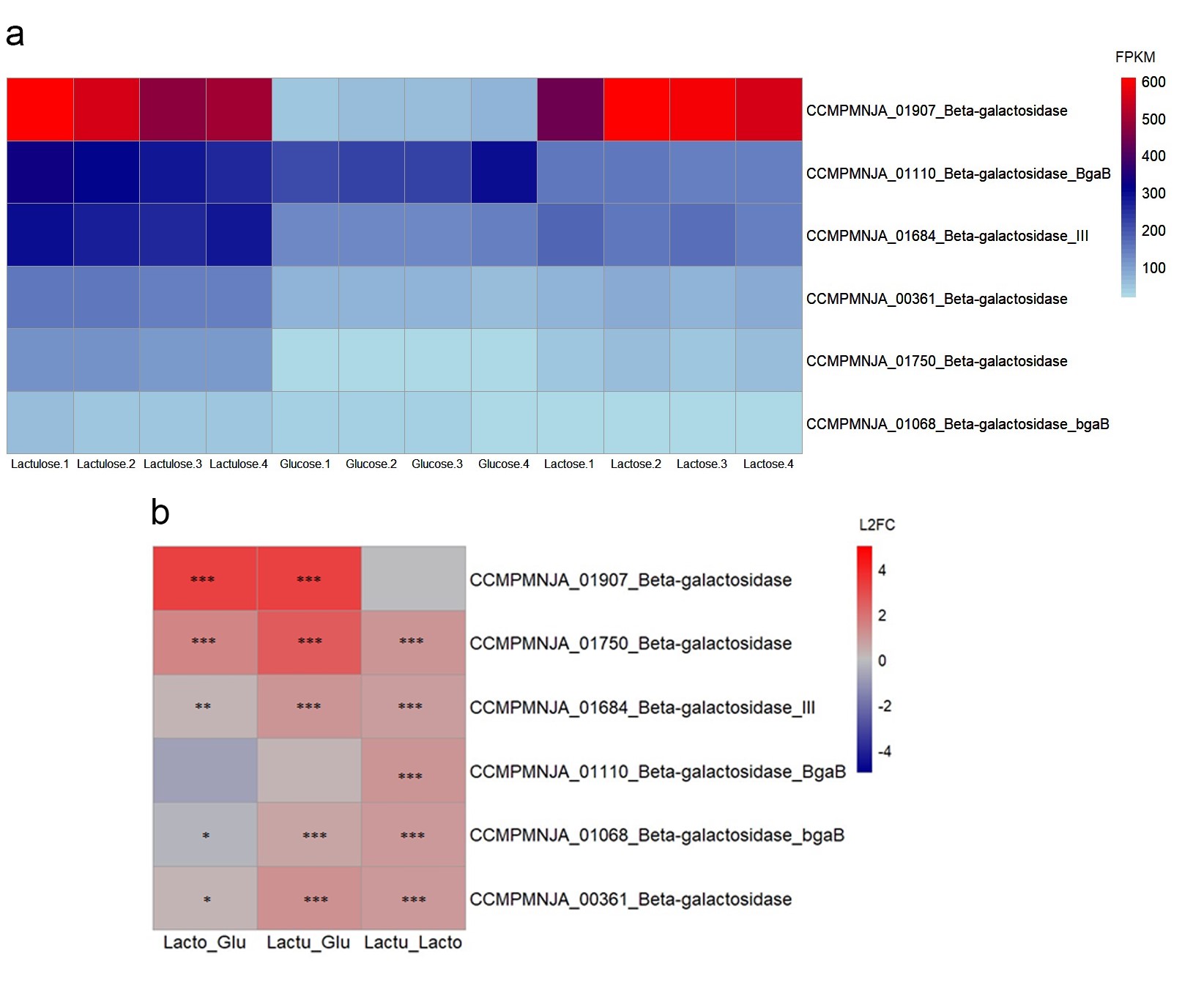


**Supplementary Figure 7. Differential Expression of β-galactosidase Genes** (a) Expression Levels of β-galactosidase Genes in *Bifidobacterium adolescentis* Under Lactulose, Lactose, and Glucose Conditions. Heatmap showing the expression levels (FPKM) of β-galactosidase genes in *B.adolescentis* grown in MRS media supplemented with Lactulose, Lactose, and Glucose. The x-axis represents four technical replicates for each condition. (b) Log2 fold change (L2FC) of β-galactosidase gene expression in *B. adolescentis* across three comparisons: Lactose vs Glucose (Lacto_Glu), Lactulose vs Glucose (Lactu_Glu), and Lactulose vs Lactose (Lactu_Lacto). Red indicates upregulation, while blue indicates downregulation. Asterisks denote significant changes based on the **Wald test (DESeq2)** (*** p < 0.05, ** p < 0.01, *** p < 0.001, n= 4).


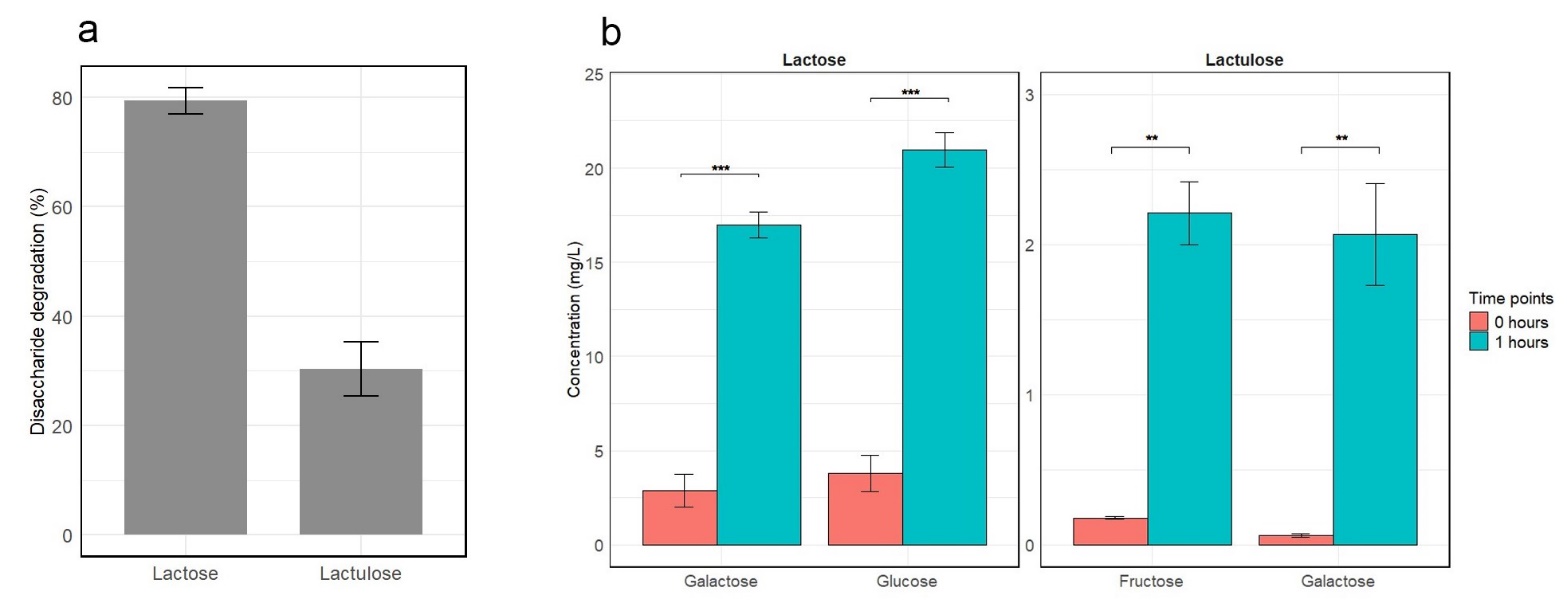


**Supplementary Figure 8. Evaluation of β-galactosidase activity.** (a) Percentage of lactose and lactulose degraded after 1 hour of incubation with commercial beta-galactosidase. (b) Detection of Monosaccharides hydrolyzed from lactose and lactulose by beta-galactosidase activity. The concentration of monosaccharides released after 1 hour of incubation with beta-galactosidase for lactose (left panel) and lactulose (right panel). The x-axis shows the detected monosaccharides (Galactose, Glucose, and Fructose), while the y-axis represents their concentration (mg/L). Red bars indicate 0 hours, and blue bars represent the values after 1 hour of incubation. A significant increase in glucose and galactose concentrations of lactose (Student’s t-test, p < 0.001, n = 6) and in fructose and galactose concentrations of lactulose (Student’s t-test, p < 0.01, n = 6) is indicated by asterisks.


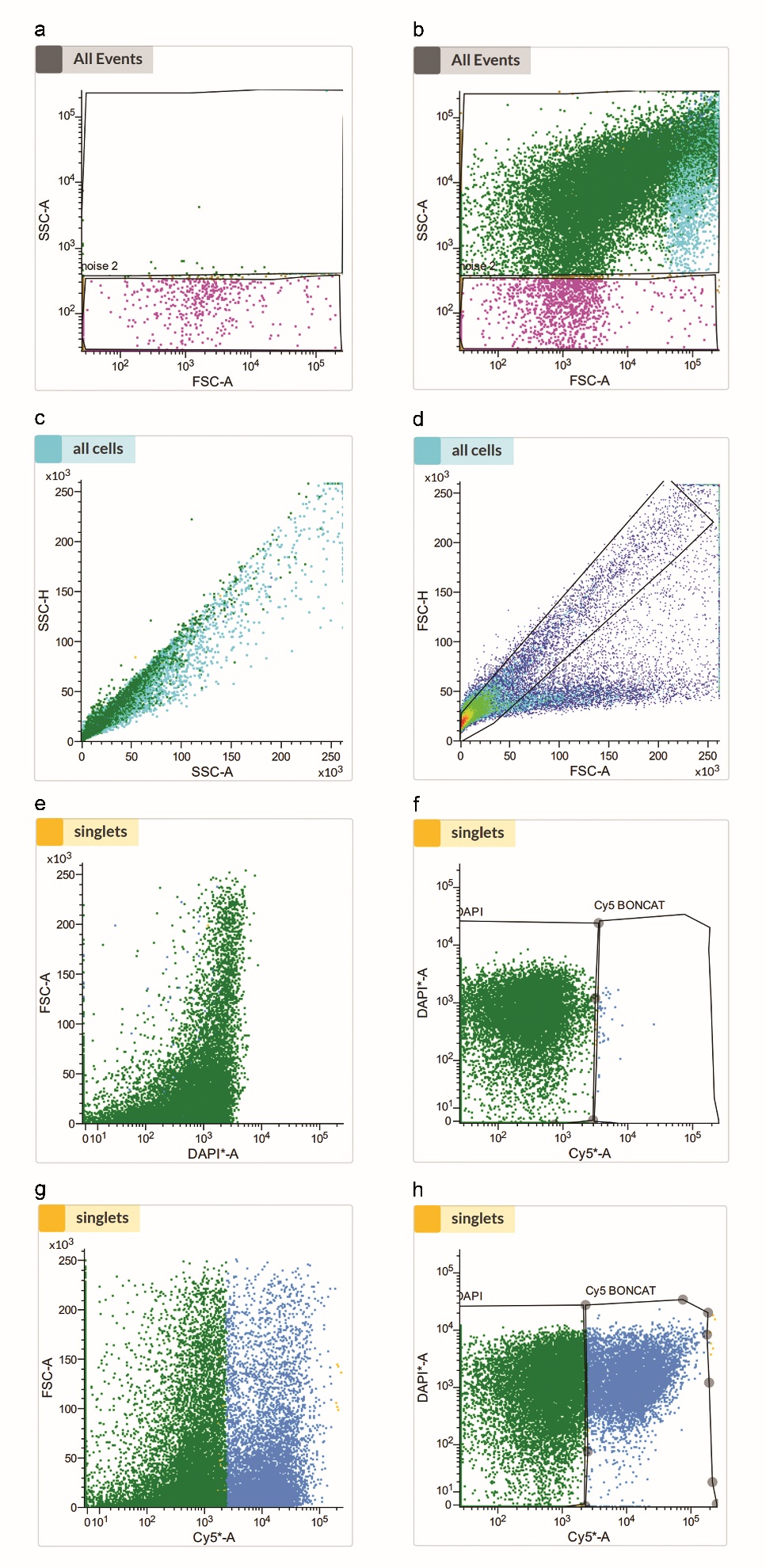


**Supplementary Figure 9**. **The full gating strategy** (a) All event: PBS blank sample. Forward scatter (FSC-A) vs. side scatter (SSC-A) plot of 1× PBS blank sample used to define background signal and set the threshold for debris exclusion. This control was used to gate out non-cellular noise from downstream cell sorting. (b) All event: sample, - DAPI, - AHA. A plot of a control sample without AHA and without DAPI staining, used to define the distribution of particles based on size and granularity and to identify background debris. (c–d) All cells: sample, - DAPI, - AHA. Doublet discrimination strategy using side and forward scatter properties of the control sample. (c) Side scatter area (SSC-A) vs. side scatter height (SSC-H) and (d) forward scatter area (FSC-A) vs. forward scatter height (FSC-H) plots were used to identify and exclude doublets. Events falling along the diagonal lines in plot were gated as single cells based on their proportional scatter signal profiles. (e–f) Singlets: sample, + DAPI, - AHA. Parent population gating strategy using control samples lacking AHA labeling. (e) Cells were visualized in a forward scatter area (FSC-A) vs. DAPI area (DAPI-A) plot to identify nucleic acid-containing events. (f) The same population was then plotted by DAPI*-A vs. Cy5*-A fluorescence, confirming the absence of BONCAT signal (Cy5-negative), which served as a baseline for setting the BONCAT-positive gate in AHA-treated samples. (g–h) Singlets: sample, + DAPI, + AHA. Gating strategy for BONCAT-labeled samples. Cells were incubated with AHA and labeled with Cy5 and DAPI to identify translationally active cells. (g) Cells were gated based on Cy5 fluorescence intensity (FSC-A vs. Cy5-A) to distinguish BONCAT-positive (blue) and BONCAT-negative (green) subpopulations. (h) A DAPI vs. Cy5-A plot was used to confirm DNA content and help separate active (Cy5⁺) from inactive (Cy5⁻) cells more clearly.
